# Data on macrophage mediated muscle transfection upon delivery of naked plasmid DNA with block copolymers

**DOI:** 10.1016/j.dib.2016.03.087

**Published:** 2016-04-01

**Authors:** Vivek Mahajan, Zagit Gaymalov, Daria Alakhova, Richa Gupta, Irving H. Zucker, Alexander V. Kabanov

**Affiliations:** a Division of Molecular Pharmaceutics, Center for Nanotechnology in Drug Delivery, Eshelman School of Pharmacy, University of North Carolina at Chapel Hill, NC 27599, USA; b Department of Pharmaceutical Sciences and Center for Drug Delivery and Nanomedicine, College of Pharmacy, University of Nebraska Medical Center, Omaha, NE 68198-5850, USA; c Department of Pathology and Microbiology, University of Nebraska Medical Center, Omaha, NE 68198-5850, USA; d Department of Cellular and Integrative Physiology, University of Nebraska Medical Center, Omaha, NE 68198-5850, USA; e Laboratory of Chemical Design of Bionanomaterials, Faculty of Chemistry, M.V. Lomonosov Moscow State University, 119899 Moscow, Russia

## Abstract

The data contains 14 figures supporting the research article “Horizontal gene transfer from macrophages to ischemic muscles upon delivery of naked DNA with Pluronic block copolymers” [1]. The data explains the surgical procedure and histological characterization of Murine Hind Limb Ischemia. The data also shows the kinetics of luciferase gene expression, spread of GFP expression through muscle and the colocalization of GFP with cellular markers in ischemic muscles injected with pDNA alone or pDNA/Pluronic. Finally the data shows the effect of Pluronic Block Copolymer to enhance total gene expression (cmv-promoter driven luciferase gene) in coculture of DNA transfected MØs with muscle cells.

## Specifications Table

**Table:** 

Subject area	*Biology*
More specific subject area	*Nanomedicine, Muscle transfection*
Type of data	*Image (microscopy), graph.*
How data was acquired	*Confocal Microscope, in vitro coculture assay.*
Data format	*Analyzed*
Experimental factors	*Coculture assay; DNA transfected macrophages* (MØs) *were cocultured with Myotubes and treated with Pluronic, Immunohistochemistry; the muscle tissue injected with pDNA alone or pDNA/Pluronic was isolated, sectioned, stained with appropriate antibodies and imaged using confocal microscope.*
Experimental features	*See experimental details for each figure*
Data source location	*The United States*
Data accessibility	*Data is with this article.*

## Value of the data

• Identifying the potential of inflammatory cells (MØs) in gene transfer to muscle fibers during inflammatory response will allow development of new materials and methodologies to further enhance the process and improve the muscle transfection efficiencies for successful clinical use in gene therapy applications.
• The data explains an in vitro model of muscle transfection using coculture of MØs and muscle cells to study gene transfer.
• The data shows unidirectional exchange of membrane markers and GFP from MØs to adjacent muscle cells upon coculture.
• The experimental methodologies explained can be used to study the effect of other immune cells or polymer/lipids on skeletal muscle transfection.

## 1 Data

Data shows increase in gene expression in ischemic muscles than healthy muscles upon direct administration of pDNA or pDNA/Pluronic (Fig. 2). The gene expression colocalized with inflammatory cells (MØs) and the spread of overall GFP expression was higher in ischemic muscles injected with pDNA/block copolymer than naked pDNA alone (Fig. 3, Fig. 4, Fig. 5). Coculture of transfected MØs with muscle cells shows gene transfer across cell membranes resulting in GFP^+^ muscle cells (Fig. 8) and enhanced total gene expression in coculture which further increase upon treatment with block copolymers (Fig. 10, Fig. 12, Fig. 13)**.**

## 2 Experimental design, materials and methods

### 2.1 Murine Hind Limb Ischemia Model (MHLIM)

**Fig. 1:**
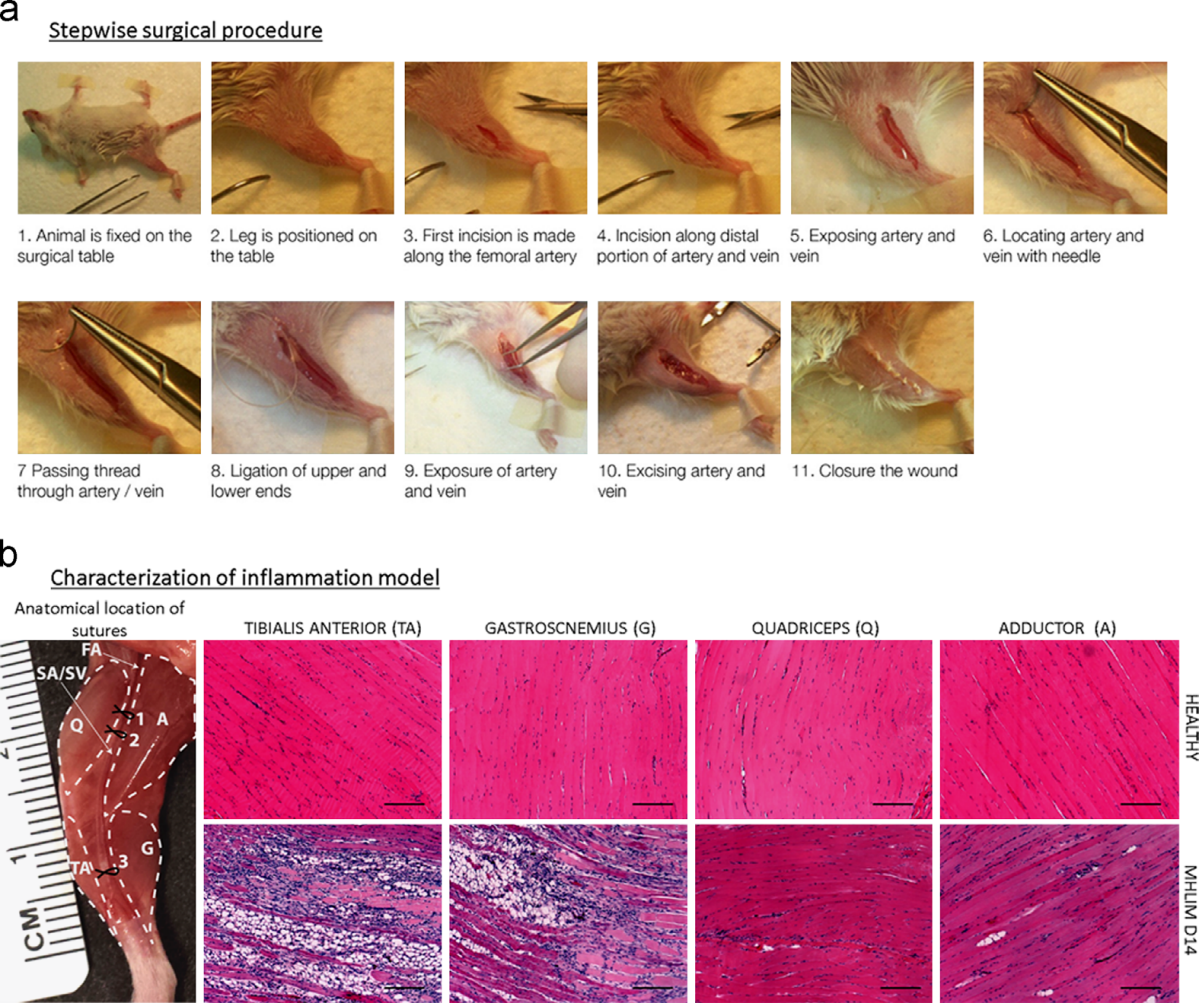
Stepwise surgical procedure and characterization of inflammation model (MHLIM). (a) Animals were anesthetized and surgical removal of artery and vein was performed as shown in (b – left) on right leg. (b) Anatomical and histological representation of ischemic hind limb muscle tissues and sections. Skin and fascia were removed to show the right hind limb muscles of balb/c mice and the sites of ligation during surgical procedure. Hind limb ischemia was generated by excision of *FA* before femoral bifurcation (between sutures marked 1 and 2) and excision of both saphenous artery and saphenous vein (between sutures 2 and 3). *TA*, G, Q and A: designate different muscles analyzed. H&E stained 5 µm tissue sections of healthy muscles (top row) and ischemic muscles at 14th day post ischemia surgery (bottom row) show histopathology of ischemia with a typical inflammatory response (cellular infiltrate) during an inflammation. Tissue sections were imaged using 10× objective magnification and images are representative of 5 slides per tissue and 3 animals per group. Consistent with previous reports [2], the effect of ischemia was more pronounced on lower hind limb muscles (TA and G) compared to upper hind limb muscles (Q and A). Similar data was obtained H&E sections of ischemic muscles at 3rd day post ischemia surgery (not shown). Scale bar 100 µm.

MHLIM was generated as described in materials and methods of Ref. [1] and shown in a stepwise surgical procedure in Fig. 1a. Muscle tissues [quadriceps (Q), adductor (A), gastrocnemius (G) and *(TA*)] at specified time points were removed en bloc, processed for H&E staining as described earlier and the tissue sections were imaged by bright field microscopy to show inflammatory cell recruitment response in MHLIM (Fig. 1b).

### 2.2 Luciferase activity *in vivo*

**Fig. 2:**
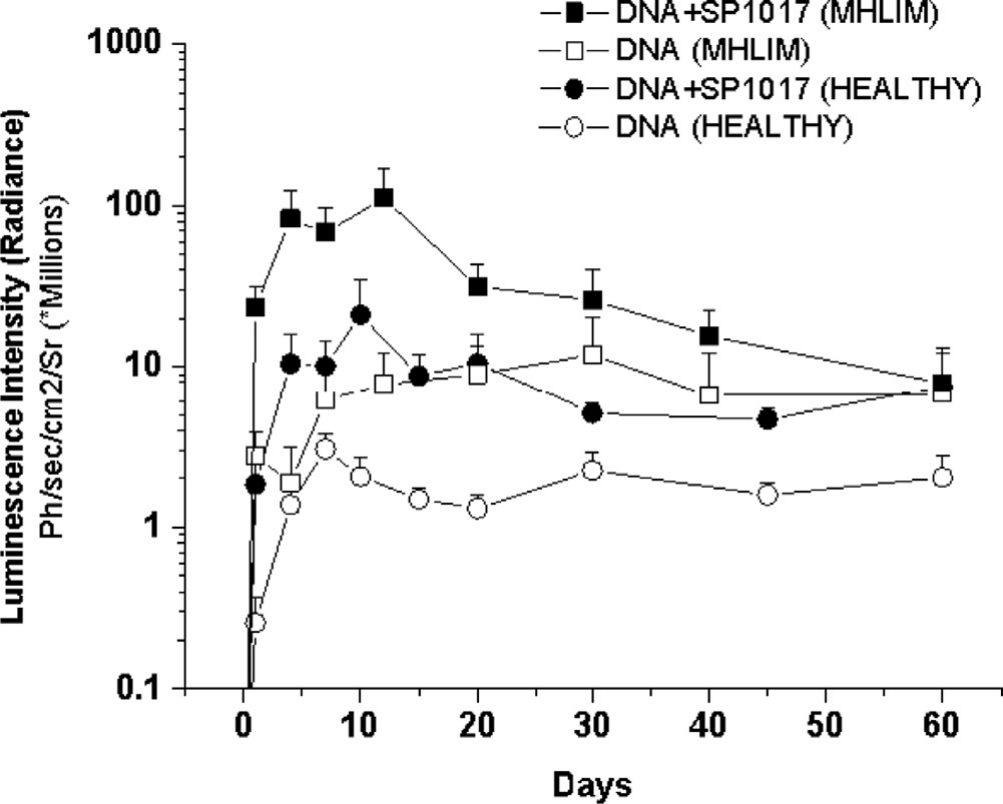
Kinetics of gene expression. Time course of luciferase expression determined in live animals by quantifying the bioluminescence imaging data (IVIS 200) for healthy and MHLIM mouse after a single injection of 10 µg naked pDNA (gWIZ™ Luc, 1st and 2nd row) and pDNA formulated with 0.1% w/v SP1017 (3rd and 4th row) in 50 µl HBSS solution. Data are mean±SEM, *n*=3–4 for each treatment group and AUC quantified each single animal and analyzed as presented in Fig. 1e in Ref. [1].

Muscles were injected with pDNA alone or pDNA/Pluronic as described in Ref. [1]. Unless indicated otherwise mice were euthanized at the time points indicated in the and tissues were processed. The luciferase activity in 10 µl tissue homogenates was quantified using a TD20/20 (Promega, Fitchburg, WI) for an integration period of 10 s and normalized per mg of tissue as described before [3]. Alternatively, luciferase gene expression was measured in live animals using *in vivo* imaging system IVIS-200 (Xenogen Corporation, Alameda, CA) 5 min after *i.p* injection of D-luciferin, the imaging data were quantified as described before [1], [3] and shown in Fig. 2. Briefly, 100 µl of D-Luciferin (150 mg/kg) was delivered by i.p. injection. Equal sized circular region of interest (ROI) were positioned to capture the signal in the DNA injected muscles for each mouse and the absolute signal of each muscle was determined at each time point as Photons/s/cm^2^/Sr.

### 2.3 Immunohistochemistry (IHC), immunocytochemistry (ICC) and confocal imaging

**Fig. 3:**
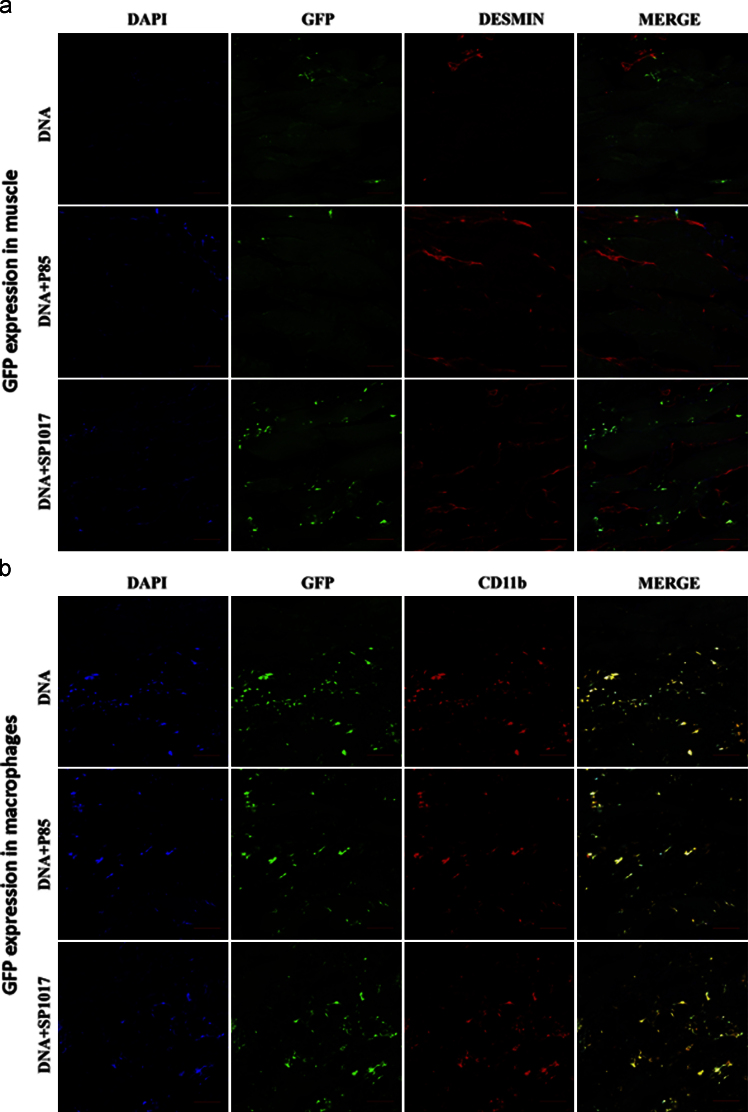
GFP expression and colocalization with cellular markers: (a, b) Colocalization of GFP expression with muscle (a) and MØ (b) markers visualized in longitudinal sections of ischemic *TA* muscles 4 days after gWIZ™ GFP injections. The color staining corresponds to nucleus (blue); MØs (CD11b^+^; red); myocytes (desmin^+^; red); and GFP (green). The last panels in each row present digitally superimposed images of preceding panels to visualize the colocalization (yellow). The images were taken with Zeiss 710 confocal laser scanning microscope using 20× objective, scale bar 50 µm.

**Fig. 4:**
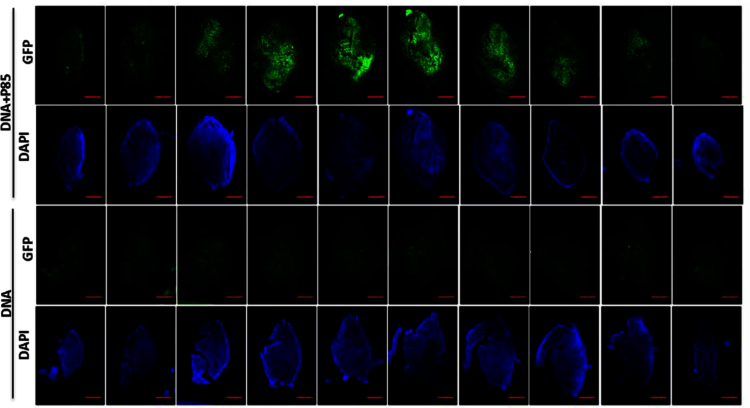
GFP expression through ischemic muscle tissue. Tile scanning confocal microscope images (10×) of 20 µm thick cross-sections at every 500 µm throughout the whole *TA* muscle tissue 4 days after injections of gWIZ™ GFP pDNA alone or pDNA with 0.6% w/v P85 in ischemic muscles in MHLIM. Representative images from each treatment group with *n*=3 are shown. Scale bar=1 mm.

**Fig. 5:**
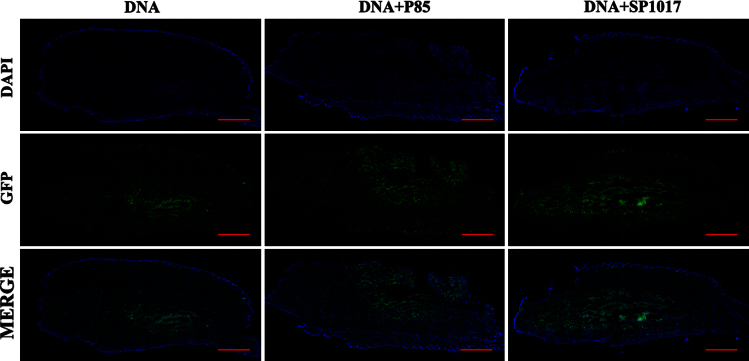
Spread of GFP expression in ischemic muscles. 4 days after pDNA injections in ischemic muscles, the spread of GFP expression in 20 µm thick longitudinal tissue section was visualized by tile scanning using confocal imaging (10×). Scale bar=1 mm. The muscle specimens were harvested after injection and individually processed for IHC. Whole muscle longitudinal sections were imaged by confocal tile scanning to view the overall GFP expression. Pictures are representative of 3 slides per group.

Muscles injected with pDNA or pDNA/Pluronic were isolated and gene expression was analyzed using IHC. Fresh muscle tissues were embedded in Tissue-Tek OCT (Sakura Finetec Inc, Torrance, CA), rapidly cooled to −80 °C and sectioned with cryostat microtome. 10 μm thick sections were attached to Superfrost^®^ microscope slides (Fisher Scientific, Bellefonte, PA), dried for 1 h at RT and stored at −80 °C for subsequent use. Double staining immunofluorescence was performed in the frozen muscle tissue sections to determine cell types expressing GFP. The sections were sequentially treated with (a) polyclonal rabbit anti-desmin antibody (Abcam, Cambridge, MA) 1:100, Rat anti-CD11b (eBioscience, San Diego, CA) 1:100 antibodies and then (b) with fluorophore-conjugated secondary anti-species antibodies (Goat anti rat-Alexa 594/Goat anti rabbit-Alexa 633) 1:1000. Specifically, frozen sections were incubated at RT for 10–15 min and fixed/permeabilized in ice cold methanol for 5 min, followed by ice cold PBS rinse (twice). Slides were incubated with 10% normal goat serum in 1× PBS (blocking solution) for 1 h at 4 °C, rinsed with PBS (thrice) and incubated with primary antibody in 2% blocking solution o/n at 4 °C. After rinsing with PBS (thrice), the slides were incubated with secondary antibodies in 2% blocking solution for 1 h at RT. Finally, the slides were counterstained with DAPI using wet mounting system (Vectashield, Burlingame, CA), stored in 4 °C until examined under microscope. Negative control specimens (treated with secondary antibody alone) were used for setting confocal lasers. The samples were analyzed by Zeiss 710 Confocal Laser Scanning Microscope equipped with a blue diode 405 nm (nucleus), argon laser 488 nm (GFP expression), DPSS 594 nm (cell marker) and HeNe 647 nm (cell marker) using 10× or 20× objective. The data shows colocalization of GFP expression with CD11b and desmin markers (Fig. 3). In another experiment the spread of GFP expression through pDNA injected ischemic muscles was analyzed using tile scanning confocal imaging as shown in Fig. 4, Fig. 5.

### 2.4 Plasmid DNA uptake in cells

**Fig. 7:**
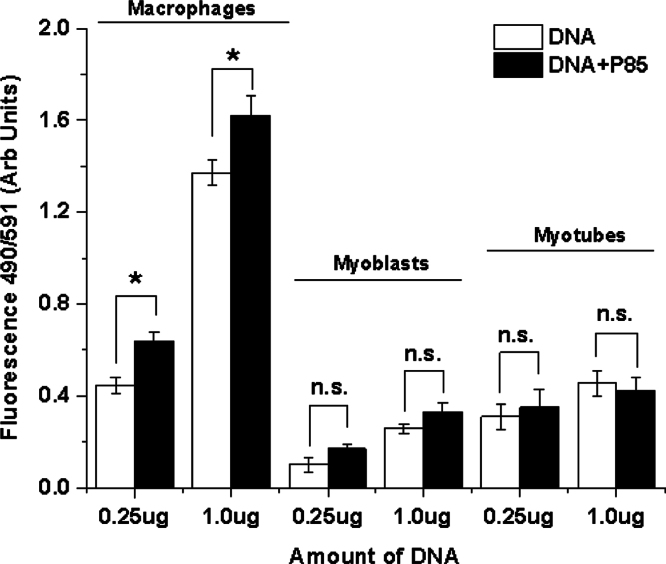
Effect of Pluronic on pDNA uptake in various cell types *in vitro*. RAW 264.7 MØ, C2C12 MBs and C2C12 derived D7 MTs (50,000 cells/well in 96 well plates, 24 h after plating) cells were exposed to YOYO-1 labeled gWIZ^™^ Luc pDNA (0.25 µg and 1 µg) in the absence (white bars) or presence of 1% w/v P85 (black bars) in SFM for 2 h. After that cells were rinsed thrice with PBS and lysed using 50 μl M-PER^®^ cell lysis reagent (ThermoFisher Scientific, Vernon Hills, IL) for 5–10 min at 4 °C. Total fluorescence was quantified in cell lysates using Spectramax M5 plate reader (Molecular devices, Sunnyvale, CA) at λ Ex/Em 490/591. Data are mean±SEM (*n*=6). Statistical comparisons were made by Pair-wise *t*-test with Welch׳s correction: *p<0.05, n.s. – non significant.

50,000 cells/well (RAW 264.7 MØs, C2C12 MBs and C2C12 derived MTs) were plated in 96 well plate a day before experiment. DNA was labeled with YOYO-1 dye as described earlier [4] and incubated with or without 1.0% (w/v) P85 solution for in SFM. After 2 h cells were rinsed thrice with PBS and lysed using 50 μl MPER cell lysis solution (Thermo Fisher Scientific, Rockford IL) for 5–10 min at 4 °C. Total fluorescence was quantified in cell lysates using Spectramax M5 plate reader (Molecular devices, CA) at λ Ex/Em 490/591 nm and data is shown in Fig. 7.

### 2.5 *in vitro* GFP transfer from transfected MØs to muscle cells (MBs)

**Fig. 8:**
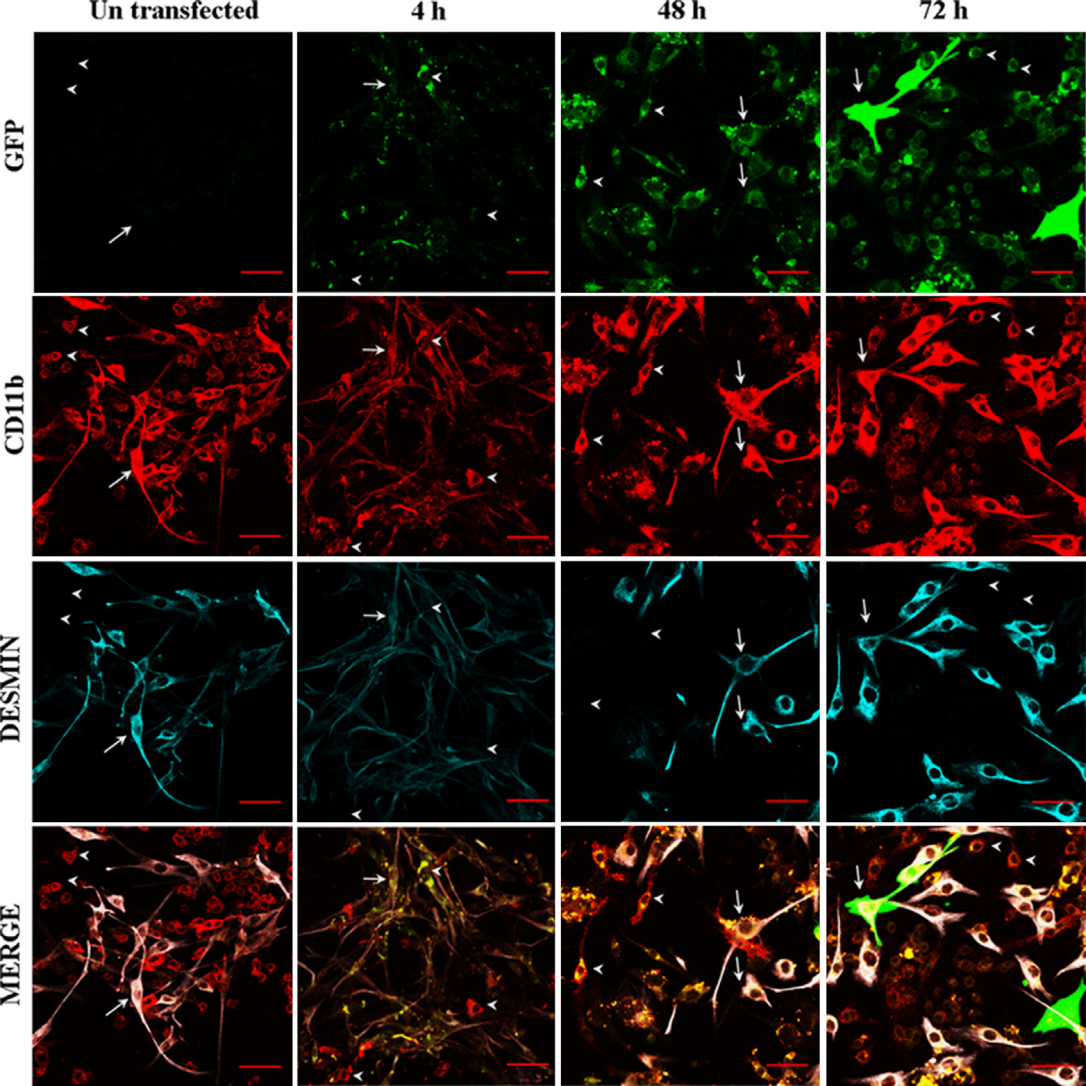
*in vitro* transfection of muscle cells upon coculture with GFP transfected MØs. MØs (arrow heads) were transfected with gWIZ™ GFP pDNA and then cocultured with un-transfected MBs (arrows) for up to 72 h. At specific time points cells were harvested, rinsed twice with ice cold PBS, fixed with ice cold methanol, processed for blocking and labeling with primary and secondary antibodies as explained above in IHC. The control coculture (untransfected MØs) was imaged at 48 h. MBs stained positive for both GFP and CD11b at each time point. The color staining corresponds to GFP (green), CD11b (red), and desmin (cyan). The bottom panels present digitally superimposed images (20×) of preceding panels to visualize the co-localization (yellow or white). Scale bar=50 µm.

**Fig. 9:**
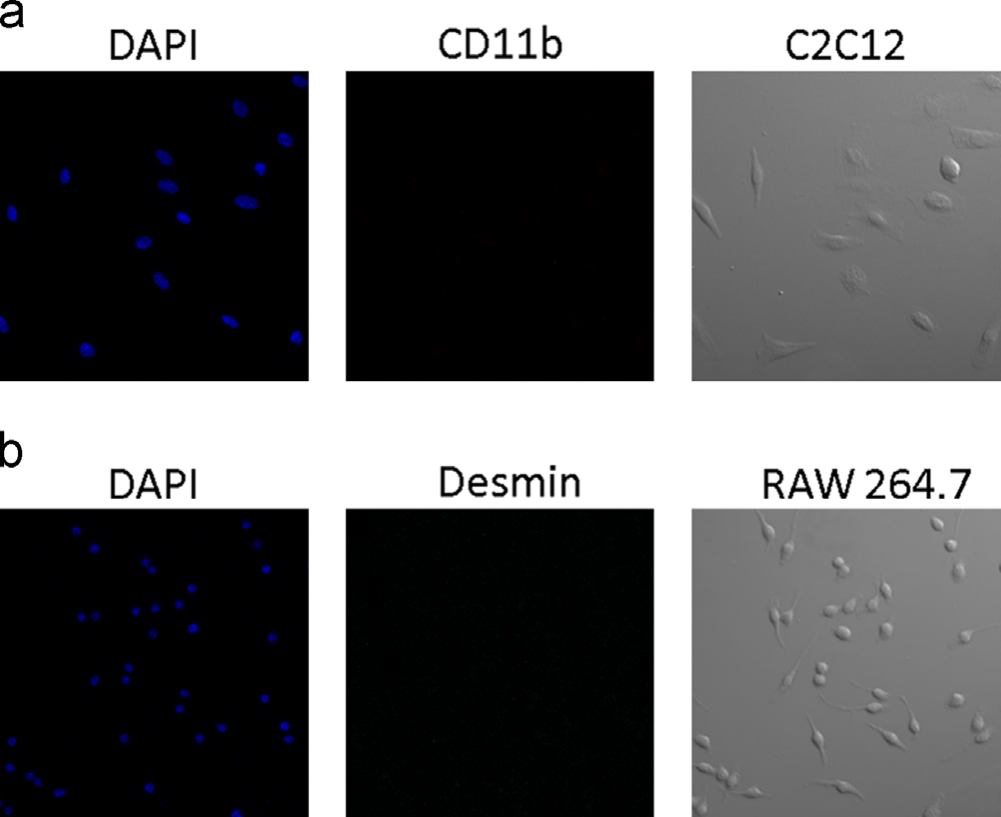
Lack of cross-reactivity between anti-desmin and anti-CD11b antibodies in MBs and MØs cultured separately. (a) MBs and (b) Raw 264.7 MØs were labeled with anti-CD11b and anti-desmin antibodies respectively to confirm the non cross reactive nature and to rule out false positive staining in the coculture experiments.

To further examine GFP transfer from MØs to muscle cells at the cellular level, we developed an in vitro coculture model. Briefly, gWIZ™ GFP transfected MØs were co-cultured with MBs, and GFP expression in MØs and MBs was visualized by confocal imaging at different time points. As shown in Fig. 8, already at 4 h coculture MBs stained positive for MØ marker (CD11b) and not *vice versa* implying a unidirectional transfer of membrane components from MØs to MBs upon coculture. Without coculture there was no staining of MBs with CD11b or MØs with desmin (Fig. 9). At the 4 h time point in Fig. 8 the arrowheads point to a CD11b^+^ and desmin^−^ MØs one of which express GFP and another does not. Notably, the transfection is heterogeneous, with only 20–30% of cells being transfected initially. At the same time point the arrow points to a CD11b^+^ and desmin^+^ MBs with low or no level of GFP expression. At the 48 h and 72 h time points along with CD11b^+^ and desmin^−^ GFP-expressing MØs (arrowheads) we clearly observed CD11b^+^ and desmin^+^ GFP-expressing MBs (arrows). Interestingly, by 72 h the GFP expression in MBs appears to increase while the reporter gene expression in MØs somewhat faded.

### 2.6 Gene expression in coculture

**Fig. 10:**
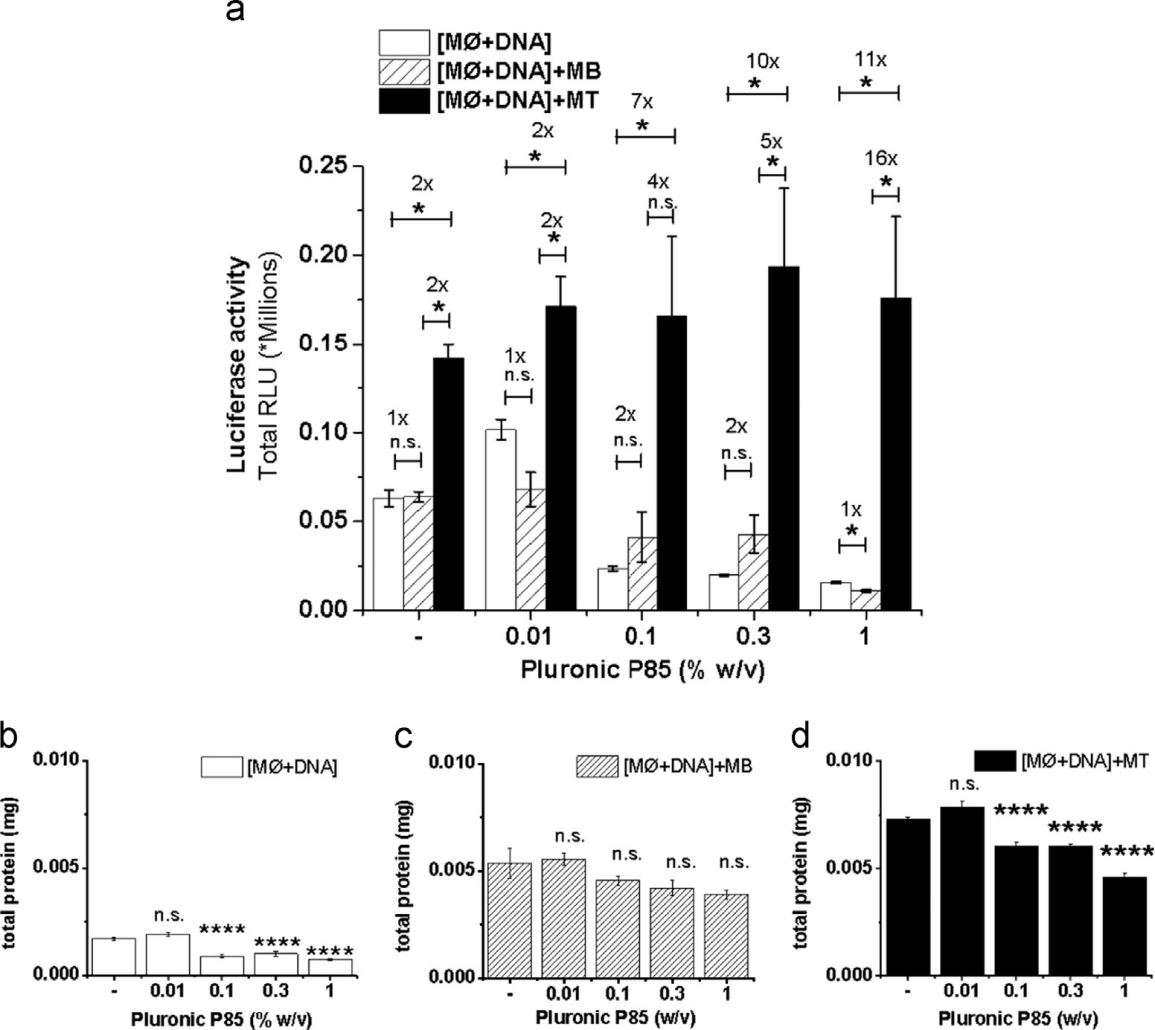
Effect of **p**luronic on total gene expression and protein levels in the transfected MØs and their co-culture with muscle cells: (a) pDRIVE5Lucia-mDesmin transfected MØs were plated alone ([MØ+DNA]), or cocultured on top of the monolayer of MBs ([MØ+DNA]+MB) or MTs ([MØ+DNA]+MT). After 2 h, when MØs attach to the MBs or MTs, the groups were treated with increasing concentrations of P85 (0.01%, 0.1%, 0.3% and 1.0% w/v) or fresh media for 2 h, washed, further incubated. The total secreted luciferase expression was analyzed after 24 h in cell culture media. Total protein content was determined in cell lysates after 24 h for (b) [MØ+DNA], (c) [MØ+DNA]+MB and (d) [MØ+DNA]+MT groups with and without P85 treatment. Data are mean±SEM (*n*=6). Statistical comparisons were made (a) using multiple t-tests with Holm–Sidak correction for multiple comparisons for treated vs. untreated groups, or (b–d) using one-way ANOVA with Bonferroni correction for multiple comparisons. *p<0.05, **p<0.005, n.s. – non significant.

**Fig. 11:**
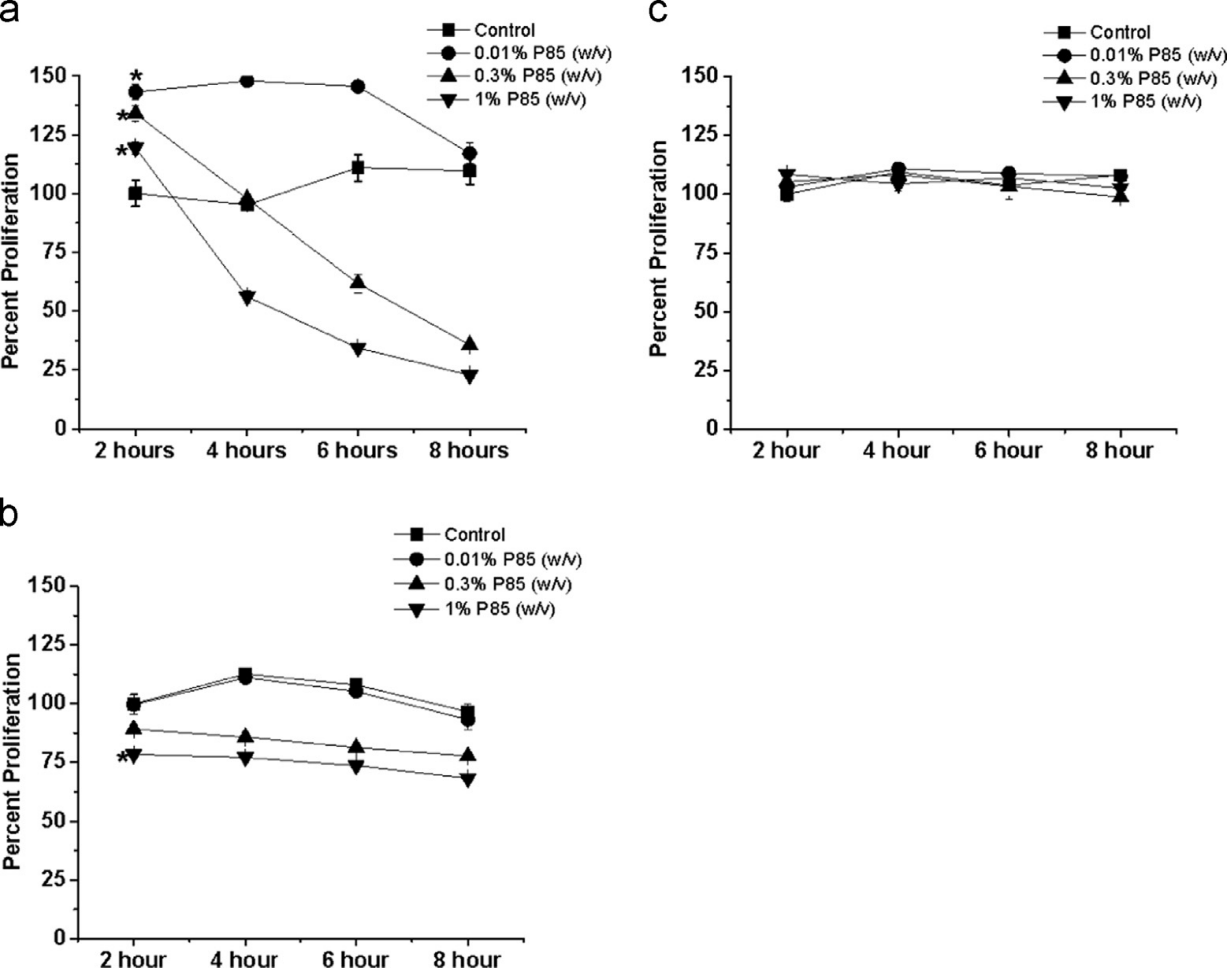
Cytotoxicity of P85 on MØs (a), MBs (b) and terminally differentiated MTs (c) was determined after exposure of the cells to different concentrations of P85 (0.01%, 0.3% and 1.0%) for different durations (2, 4, 6, and 8 h) in SFM. Cells were further cultured for total 24 h if fresh media and the percent cell proliferation, i.e. treated cells compared to untreated controls, was determined by MTS assay. Data are mean±SEM (*n*=6); cell viabilities are compared at 2 h time point using unpaired t-test with Welch׳s correction: *p<0.05, n.s. – non significant. At all-time points examined the proliferation of MØs exposed to lower concentrations of P85 (0.01% w/v) was significantly increased compared to untreated controls. Likewise MØs exposed to higher concentrations of P85 (0.3% and 1.0% w/v) for 2 h exhibited greater proliferation compared to untreated controls. However, at longer exposures (4, 6 and 8 h) these relatively high concentrations of P85 induced cytotoxic effect in MØs. Exposure of MBs to P85 at higher concentrations of (0.3% and 1.0% w/v) induced some limited cytotoxicity. MTs were the most resistant cells with respect to P85 as no toxicity was observed at any concentration of P85 at any time point.

**Fig. 12:**
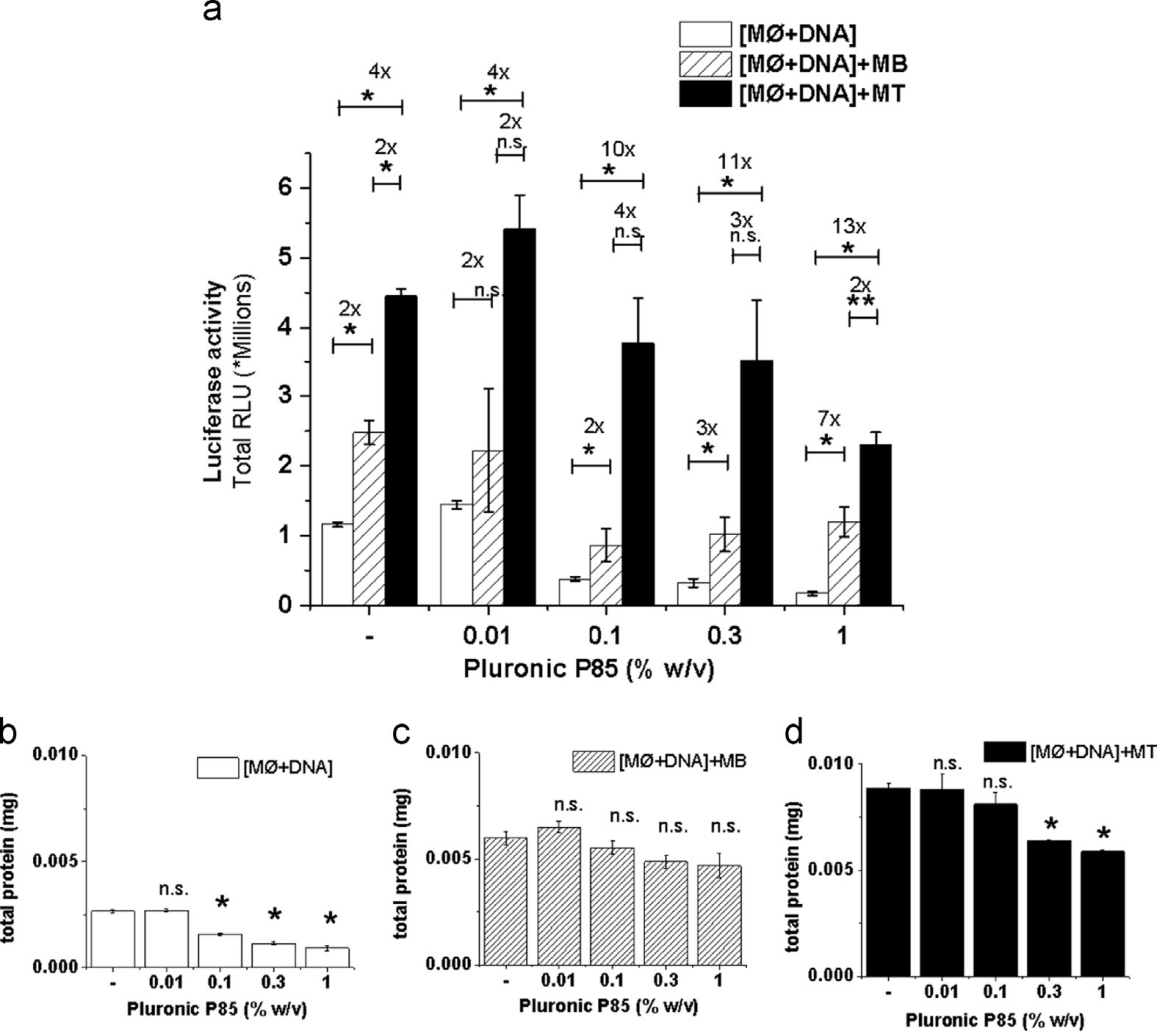
Effect of pluronic on total gene expression and protein levels in the transfected MØs and their co-culture with muscle cells: (a) gWIZ™ Luc pDNA transfected MØs were plated alone, [MØ+DNA], or cocultured on top of the monolayer of MBs, [MØ+DNA]+MB, or MTs, [MØ+DNA]+MT. After 2 h, when MØs attach to the MBs or MTs, the groups were treated with increasing concentrations of P85 (0.01%, 0.1%, 0.3% and 1.0% w/v) or fresh media for 2 h, washed, further incubated. The total luciferase expression analyzed after 24 h in cell lysates. Total protein content was determined in cell lysates after 24 h for (b) [MØ+DNA], (c) [MØ+DNA]+MB and (d) [MØ+DNA]+MT groups with and without P85 treatment. Data are mean±SEM (*n*=6), Statistical comparisons were made (a) using multiple *t*-tests with Holm–Sidak correction for multiple comparisons for different co-cultures at a given Pluronic concentration, or (b–d) using one-way ANOVA with Bonferroni correction for multiple comparisons. *p<0.05, **p<0.005, n.s. – non significant.

**Fig. 13:**
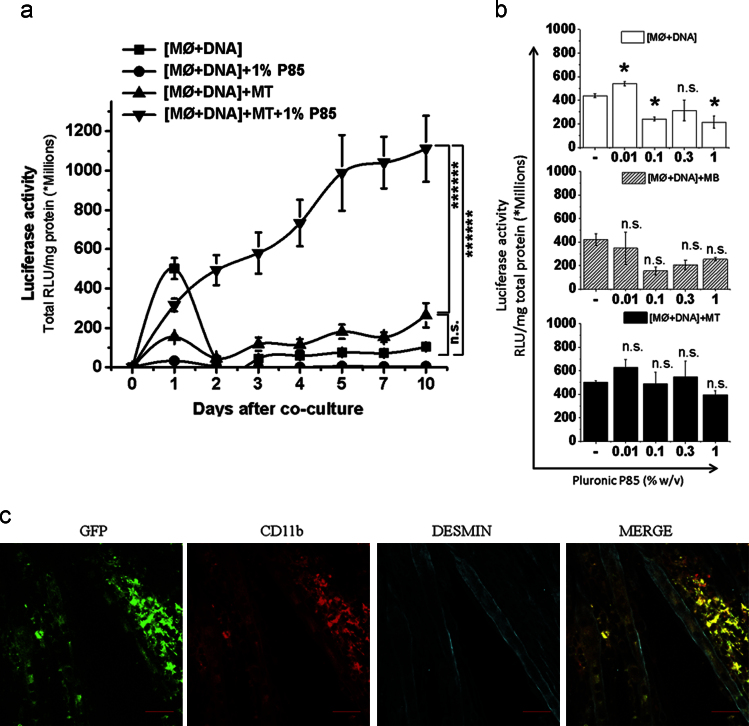
Effect of P85 on horizontal gene transfer from transfected MØs to muscle cells upon co-culture. (a, b) gWIZ™ Luc pDNA transfected MØs were plated alone, [MØ+DNA], or cocultured with MBs, [MØ+DNA]+MB, or MTs, [MØ+DNA]+MT, and exposed to P85 (0.01%, 0.1%, 0.3% and 1.0% (b) or 1% w/v (c)) for 2 h. The total luciferase in cell lysates was determined (a) daily for 10 days or (b) after 24 h and normalized for the cell protein. A significant decrease in gene expression in [MØ+DNA] *v.s.* [MØ+DNA]+P85 groups at day 1 can be explained by detachment of freshly plated MØs upon 1% P85 treatment, which also explains the decrease in gene expression in upon treatment with increasing concentration of P85. (a, b) Data represents mean±SEM, (a) (*n*=12), (b) (*n*=6). (a) The AUCs for each individual condition were calculated and compared using one-way ANOVA with Bonferroni correction for multiple comparisons. (b) Statistical comparisons were made using one-way ANOVA with Bonferroni correction for multiple comparisons * *p*<0.05, n.s. – non significant (c) GFP expression (green) in desmin^+^ MTs (cyan) was validated 3 days after their coculture with MØs CD11b^+^ (red) transfected with gWIZ™ GFP pDNA. The last panels in each row present digitally superimposed images (20×) of preceding panels to visualize the co-localization (yellow). Scale bar=50 µm.

**Fig. 14:**
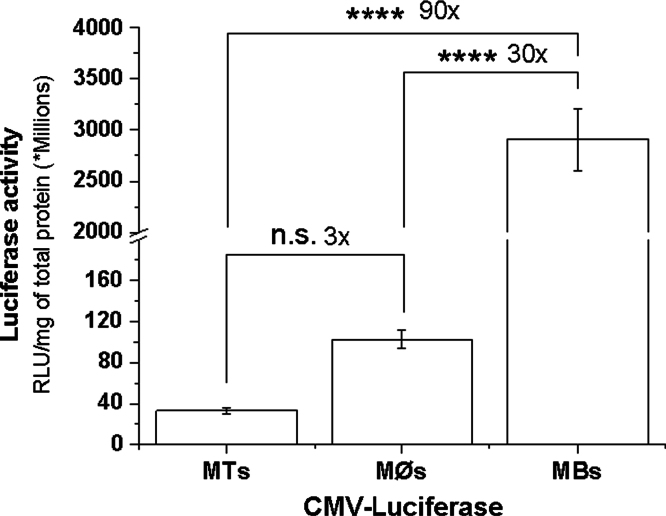
Relative transfection efficiencies in MØ, MB and MT *in vitro**.*** Terminally differentiated C2C12 derived MT, precursor C2C12 MB and RAW264.7 MØ were transfected with gWIZ™ Luc (cmv-luciferase) using genePORTER300 and gene expression was determined in cell lysates after 24 h. Data are mean±SEM (*n*=4); statistical comparisons were made using one-way ANOVA with Bonferroni correction for multiple comparisons: ***p<0.005, ****p<0.0001, n.s. – non significant. MTs in general were the most difficult to transfect cells. The transfection of these cells using cmv- and desmin-luciferase pDNA as 90 and 40 times less respectively compared to their precursor MBs cells. Consistent with the literature MØs were in general more difficult to transfect cells than undifferentiated proliferating cells (MBs).

To evaluate horizontal gene transfer, DNA transfected MØs were cocultured with muscle cells (MBs or MTs) and muscle specific (desmin promoter) gene expression was analyzed after 24 h as described in “materials and methods” in Ref. [1] and data shown in Fig. 10. The coculture was and treated non-toxic concentrations of Pluronic (0.01%, 0.1%, 0.3% and 1.0%) as shown in Fig. 11. Similarly, total or constitutive (cmv promoter) gene expression was also quantified in coculture model and data shown in Fig. 12, Fig. 13. Muscles tissue are relatively difficult cells to transfect in vivo. To reflect this, myotubes (invitro model of muscle) used in this study were validated by comparing the relative ability of plasmid transfection in comparison to their precursor cells (myoblasts) and macrophages (Fig. 14).

## Figures and Tables

**Fig. 6:**
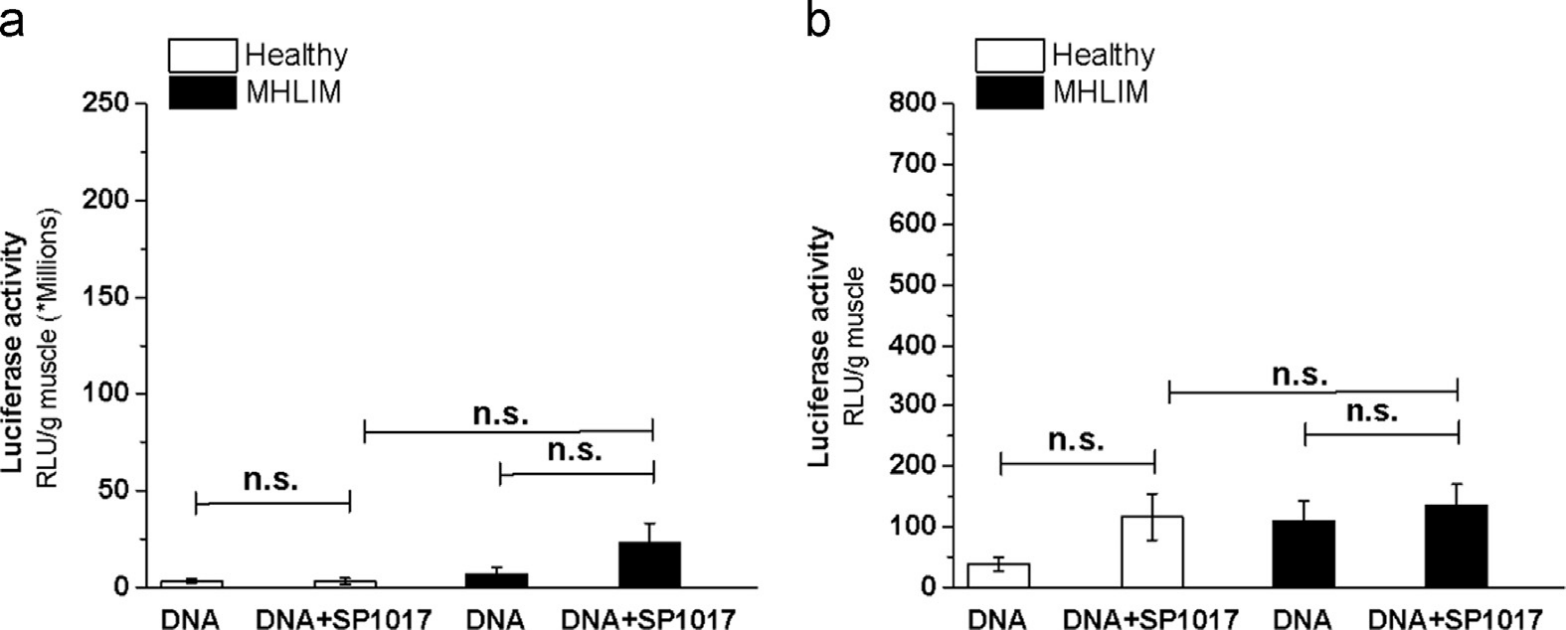
Effect of skeletal muscle ischemia on transgene expression in contralateral muscles. As per scheme in Fig. 6b in Ref. [1] the test articles were injected in *TA* muscles in healthy mice or in contralateral *TA* muscles simultaneously with the ipsilateral ischemia surgery in MHLIM. Luciferase gene expression in the (a) *TA* muscles and (b) draining lymph nodes of MHLIM (black) and healthy mice (white) was determined 3 days after single administration of 10 µg gWIZ™ Luc with or without 2.25% w/v SP1017. pDNA injections were performed in *TA* muscle of healthy mice (white bars) and healthy or non-ischemic muscles of MHLIM (black bars). Data are mean±SEM (*n*=10), n.s. – non significant. Statistical comparisons were made using one-way ANOVA with Bonferroni correction for multiple comparison.
